# Renal and Genitourinary Ultrasound Evaluation in Emergency and Critical Care: An Overview

**DOI:** 10.3390/healthcare12131356

**Published:** 2024-07-07

**Authors:** Daniele Orso, Daniele Peric, Carmine Cristiano Di Gioia, Irene Comisso, Tiziana Bove, Alessio Ban, Federico Fonda, Nicola Federici

**Affiliations:** 1 Department of Emergency “Santa Maria della Misericordia”, University Hospital of Udine, Azienda Sanitaria Universitaria Friuli Centrale, 33100 Udine, Italy; irene.comisso@asufc.sanita.fvg.it (I.C.); tiziana.bove@uniud.it (T.B.); nicola.federici@asufc.sanita.fvg.it (N.F.); 2 Department of Emergency, University Hospital of Trieste, Azienda Sanitaria Universitaria Giuliano-Isontina, 34128 Trieste, Italy; daniele.peric@asugi.sanita.fvg.it; 3 Department of Emergency Medicine, Community Hospital of Baggiovara (MO), Azienda Ospedaliero-Universitaria di Modena, 41125 Modena, Italy; digioia.cristiano@aou.mo.it; 4 Department of Medicine (DME), University of Udine, 33100 Udine, Italy; 5 Department of Pediatrics, Community Hospital of Latisana (UD), Azienda Sanitaria Universitaria Friuli Centrale, 33100 Udine, Italy; alessio.ban@asufc.sanita.fvg.it

**Keywords:** renal ultrasound, genitourinary ultrasound, renal colic, swinging kidney, vexus, renal resistivity index, review

## Abstract

Renal and genitourinary ultrasound are fundamental resources employed by emergency and critical care healthcare providers to make prompt diagnoses and perform ultrasound-guided procedures. At the bedside, ultrasound can aid in the diagnosis of relevant pathologies, such as post-renal obstruction or kidney stones, and life-threatening conditions such as aortic dissection or hemoperitoneum. A narrative overview was performed, providing an updated review of renal and genitourinary ultrasound for emergency and critical care healthcare providers, emphasizing its advantages and the latest advances in the field. A thorough summary that can be utilized as a guide for emergency and critical care healthcare providers is presented. The daily hemodynamic management of critically ill patients involves the implementation of new protocols, such as VexUS or the evaluation of the renal resistance index. The role of ultrasound in managing acute nephropathy and genitourinary issues is increasingly crucial given its bedside availability, thus this imaging modality not only facilitates the initiation of therapeutic interventions but also provides swift prognostic insights that are vital to provide tailored patient care. As further advances in ultrasound will arise, it is important for healthcare providers to foster the use of these technologies capable of improving patient outcomes.

## 1. Introduction

Ultrasound has long been recognized as an important technique for evaluating the pulmonary and cardiovascular systems in acute medicine, especially in the Emergency Department (ED) and Intensive Care Unit (ICU) settings. Nowadays, ultrasound techniques have expanded their scope and have been studied throughout the systems of the whole human body, from head to toe [1]. The point-of-care (POC) availability of ultrasound provides an important opportunity for emergency and critical care healthcare providers, allowing the potential for both (a) a prompt diagnosis and (b) the ability to perform several ultrasound-guided procedures [2]. The field of critical care medicine has been impacted by recent advances in renal ultrasound applications. The sensitivity range of this non-invasive imaging method is now in the range of 62% to 77%, and it has a specificity of 58% to 73% for renal parenchymal diseases. It is notable that it has a high positive predictive value of 92% when microscopic examination is assumed as a reference [3].

The use of ultrasound in nephrourology dates to the 1950s, with the objective of studying the morphology of major organs. However, it was only in 1976 that the Doppler study of renal vessels was introduced into clinical evaluation [4]. In the present day, genitourinary ultrasound, which is primarily used in the radiological laboratory, is becoming a useful tool for evaluating different aspects of the critically ill patient’s bedside. In this scenario, the management of acute patient care is facilitated by the application of renal and genitourinary ultrasound to obtain vital diagnostic data that aids healthcare providers in performing diagnoses and procedures.

The purpose of this article is to provide an updated overview of renal and genitourinary ultrasound for emergency and critical care healthcare providers, emphasizing its advantages and latest advances in the field.

## 2. Materials and Methods

A narrative overview was performed [5]. The study design was chosen in order to address the topic of renal and genitourinary ultrasound in a wide form [6], thus presenting the broad perspectives of the topic [5].

Literature searches have been conducted on the following electronic databases: PubMed, Scopus, and Cumulative Index to Nursing and Allied Health Literature (CINAHL). Also, manual searches of relevant scientific society websites, textbooks, and the reference lists of included articles were performed to gather all the evidence available. Search strings were combined using the Boolean operators AND, OR, and NOT, adopting the following keywords: renal ultrasound, genitourinary ultrasound, bladder ultrasound, ultrasound procedures, ultrasound techniques, acute kidney injury, renal colic, ureteral jet, renal resistivity index. The research team included and narratively synthesized documents relevant to the review’s aims. In order to guarantee rigor in the development and reporting of the narrative overview, the quality aspects of the Scale for the Assessment of Narrative Review Articles (SANRA) were adopted [6].

## 3. Results

### 3.1. Normal Sonographic Anatomy

Renal ultrasound is usually performed with a convex probe operating at a frequency of 2.5–5 MHz [7]. The imaging is conducted in two basic orientations: coronal and transverse. This comprehensive assessment includes a morphological review of the kidney, quantification of cortical thickness, measurements of the kidney’s dimensions, evaluation of tissue echogenicity, and the detection of any pathologic alterations present [8].

The kidneys are oval organs, with a long axis diameter of 9–12 cm, a width of 5–7 cm, and a thickness of 4–5 cm. The parenchyma and cortex are 1–2 cm thick and 0.8–1 cm thick, respectively. The echogenicity of the cortex is equal to or slightly less than that of the hepatic parenchyma, while the medulla is hypoechoic. The boundary between the cortex and the medulla can be easily discerned. The renal sinus forms about half to three-quarters of the kidney and is hyperechoic and heterogeneous. The renal sinus is an adipose compartment that contains the renal hilum (artery and vein), renal calyces, and lymphatic vessels. Alterations in its echogenicity may be normal variants (e.g., renal pseudotumor or hypertrophic columns of Bertin) or pathological (e.g., neoplastic invasion, metabolic alterations, etc.) [9]. A dark liquid tubular area with a diameter of <1 cm is visible inside the sinus. This area is the renal pelvis, which is a triangular shape that can be seen posteriorly in the renal hilum and is surrounded by fat and vessels, and it is made when two-to-three major calyces are united, or seven-to-eleven minor calyces are joined [10]. Its normal echogenicity can be altered by several pathological processes, including hydronephrosis, renal lithiasis, or renal pelvic transitional cell carcinoma.

In a normal color Doppler ultrasound, the kidney blood flows from the renal hilum to the renal cortex. Four degrees of renal blood flow can be measured: grade 0, when there is no flow visible, to grade 3, which signifies normal vascularization.

### 3.2. Acute Kidney Injury

Acute kidney injury (AKI) is characterized by the rapid decline in kidney function. The typical manifestation is the accumulation of the final products of nitrogen metabolism (i.e., urea and creatinine), a decrease in urinary output, or both. The K-DIGO classification, which is characterized by three levels of increasing gravity, can be utilized to determine the degree of severity of AKI. In patients who are hospitalized, the incidence of AKI is particularly significant. It is estimated that up to almost 10% of these patients suffer from some degree of acute kidney injury. Among patients admitted to the ICU, this percentage increases to 60% [11].

Both neurohormonal mechanisms and hemodynamic factors play a role in the complex pathogenesis of AKI. The direct neurotoxic effect, low renal perfusion (such as hemodynamic shock), cardiac output deficit, or an inflammatory state are just some of the causes of AKI. Both pre-renal and intrinsic renal causes can affect the pathogenesis of AKI. The urinary flow obstruction can be a post-renal cause [11].

Despite acute tubular necrosis being the main cause of AKI, which is correlated with inflammation and hemodynamic factors, ultrasound does not play a secondary role in this scenario. The primary objective of ultrasound is to determine if there is a post-renal cause of AKI (that is, urinary outflow obstruction) [12]. Hydronephrosis is the main manifestation of urinary obstruction, and we will discuss it further.

Ultrasound can be used to suggest the timing of renal failure when there is no post-renal cause. Small kidneys (i.e., a major length ≤ 10 mm) with a thin cortical (i.e., ≤6 mm) may indicate long-lasting damage [13,14,15]. In contrast, kidneys that are enlarged and hyperechoic can indicate nephritis or acute tubular necrosis [16].

The hemodynamic evaluation related to AKI and to the condition of hemodynamic shock fully includes the interpretation of the resistive index; however, we will discuss this more extensively in the continuation of our review. For now, it is sufficient to acknowledge that an increase in the resistivity index has a significant impact on the prognosis of AKI [17].

### 3.3. Renal Colic

Renal colic—a common cause of abdominal pain often encountered by EDs—is caused by obstruction and distension of the ureter and renal pelvis, which causes a sudden, severe, and sharp pain in the side and lower back. Pain can be associated with a wide range of neurovegetative symptoms such as nausea, vomiting, and diaphoresis. Although the main cause is nephrolithiasis, renal colic can be caused by other causes of ureteral obstruction, such as ureter spasm, intra-ureteral thrombus, or obstruction caused by acute papillary necrosis. The diagnosis is based on clinical, laboratory (including microscopic hematuria), and ultrasound assessment.

The prevalence of nephrolithiasis is about 13% in developed countries [18]. In this context, some scores (such as the STONE score) can predict the likelihood of ureteral stones in patients with flank pain [19]. The STONE score should only be used when there is normal kidney function in non-febrile patients and not when there has been recent trauma or ureteral surgery.

Even with low and ultra-low radiation doses, the CT scan is still considered the gold standard imaging technique [20]. A consensus among multiple disciplines suggests that point-of-care ultrasound, which has a sensitivity of about 70% and specificity of about 75%, can aid in preventing uncomplicated patients from being exposed to ionizing radiation [21].

**Figure 1:**
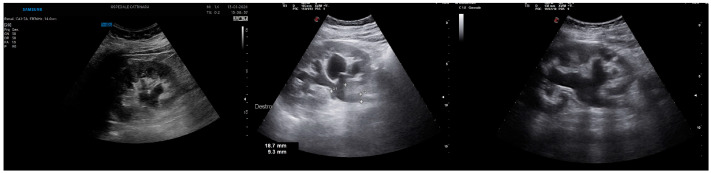
Renal hydronephrosis. From left to right: II-grade hydronephrosis. A clear dilation of the renal pelvis and a small dilation of the calyces are present. It should be noted that there are some parapelvic cysts. III-grade hydronephrosis. It is noted that although the renal calyces are dilated, they are still recognizable. IV-grade hydronephrosis. Renal calyces are difficult to identify. The pelvis and renal calyces are completely dilated. Also, the thinning of the cortex can be noted.

A unilateral dilation of a long tract of the ureter and renal pelvis is the primary ultrasound sign of renal colic (Figure 1). The severity of hydronephrosis can be classified into four degrees. The intensity of pain is correlated (at least partially) with the degree of hydronephrosis [22] and the prognosis of kidney function [23].

### 3.4. Ureteral Stone

**Figure 2:**
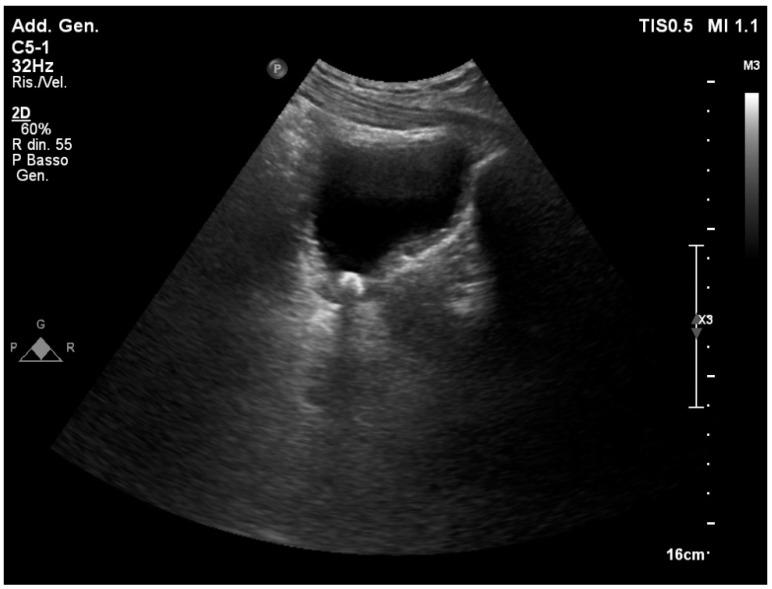
The hyperechoic calculus that produces a posterior shadow cone can be identified.

On the ultrasound, kidney stones appear as a hyperechoic structure with a shadow cone behind them (Figure 2). In addition to determining hydronephrosis and grading its severity, ultrasound has a prognostic significance in the case of nephrolithiasis. The literature agrees that the size and location of the stone are predictive factors for spontaneous expulsion [24]. If the ureteral calculus is small (having a maximum diameter of <10 mm) and distal along the ureter tract, there is a higher chance of spontaneous expulsion [25]. In addition to these factors, leukocytosis could also play an analogous prognostic role [26,27].

The size and location of the ureteral stone can be determined by expert operators using ultrasound, with a 73% agreement with the CT scan [28]. The management of patients with renal colic in the acute setting can be impacted by ultrasound. Clinical conditions (such as shock, fever, and others), the severity of hydronephrosis, and the location and size evaluation of ureteral stones through ultrasound can guide patient management.

### 3.5. The “Twinkling” Artifact

**Figure 3:**
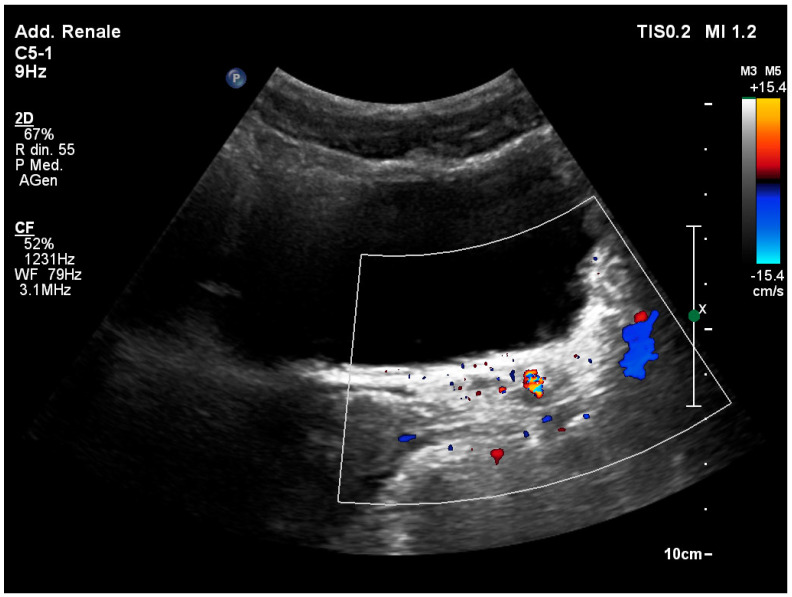
A twinkling effect is produced by a calculus located near the left ureteral meatus. The artifact is seen as a turbulent flow immediately after the calculation, as determined by the color Doppler.

The “twinkling” artifact can assist in ultrasonographic stone identification for small kidney stones, particularly those located in the juxta vesical region [29]. The “twinkling” artifact consists of a point of alternating colors in the color Doppler mode, behind a reflective object (such as a kidney stone), which takes on the appearance of turbulent flow (Figure 3). The “twinkling artifact” has a sensitivity of around 88% and a specificity of 80 to 92% in detecting kidney stones [30,31].

### 3.6. The “Swinging Kidney” Sign

The “swinging kidney” or Copetti’s sign is an ultrasound sign that has recently been discovered [32]. This sonographic sign consists of a rhythmical pitching or rocking synchronized with aortic pulsation of the kidney within a urinoma (even in minor amounts). The genesis of urinoma is due to the pressure regimes inside the Gerota capsule even in cases of low-grade hydronephrosis. Although there is only one preliminary study in the literature, the data indicate a promising correlation between this sonographic sign and the juxta vesical location and the size of kidney stones. The literature has reported that the prevalence of urinoma with ureteral stones is 3.6%. It appears that urinoma formation is more closely associated with distal and smaller stones. Patients with urinoma have higher indices of inflammation and worsened renal function [33].

The Copetti’s sign could be useful to confirm the diagnosis of nephrolithiasis in the case of low-grade hydronephrosis (or in the case of an empty bladder, which is not an uncommon event in the ED). However, although promising, further studies are needed to confirm the related pathophysiology of this new ultrasound sign and its clinical utility.

### 3.7. Identification of the Ureteral Jet

**Figure 4:**
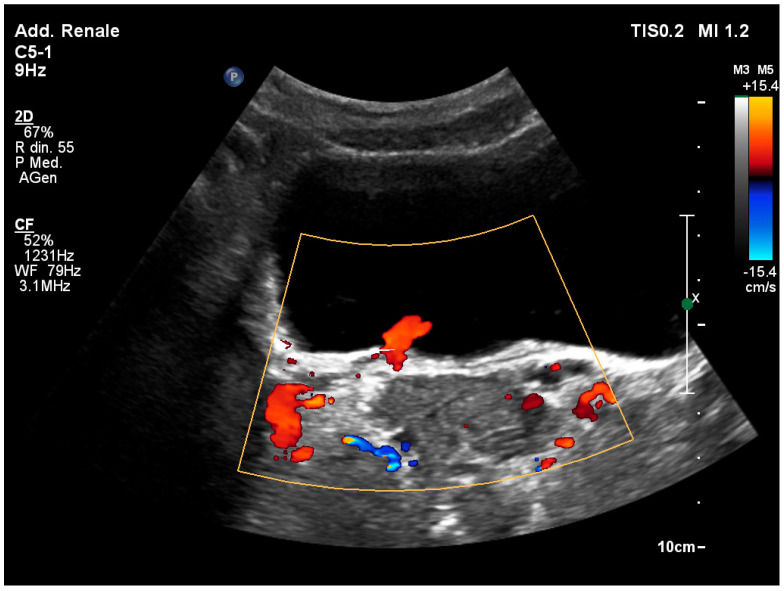
While the right ureteral jet is visible as a color Doppler stream, the left ureteral jet is not viewable.

Normally, it is possible to visualize the ureteral jet of urine from the ureteral meats inside the bladder using the color Doppler mode (Figure 4). Complete obstruction of the homolateral ureter is indicated by the absence of one of the two ureteral jets after approximately 5 min of observation. Although this finding is not specific about the cause of obstruction, it has a sensitivity range of 87% to 95% [34].

### 3.8. Ureteropelvic Junction Obstruction

Ultrasound is the leading imaging study used to diagnose ureteropelvic junction obstruction. On ultrasound, an abnormal dilatation of the pelvic system of varying degrees is observed, while the ureter is normal in caliber. Although this pathology has been primarily seen in pediatric patients, it can also be observed in adult patients [35]. The genesis is not fully understood, but it could have an intrinsic cause (such as smooth ureteral muscle agenesis) or an external cause (such as external obstruction by a vessel) [36]. This finding has the potential to enhance the collector system’s compliance in the event of higher pressure inside the tubular cells. Without causing any damage to the kidneys, most cases resolve spontaneously. To avoid unnecessary therapeutic interventions, it is crucial to differentiate between true obstruction and urinary tract dilation. The normal appearance of the renal parenchyma and the preserved size of the kidney, along with bilateral pelvic dilation and a renal index within normal limits, may indicate a non-obstructive cause [35].

In some cases, a ureteropelvic junction obstruction syndrome may be concomitant with some extra-renal diseases. There are reports in the literature associated with large inguinal hernias causing ureteral compression [37] or associated with nutcracker syndrome with extrinsic renal artery stenosis [38].

### 3.9. Acute Renal Infection

Acute kidney infections are mostly caused by pyelonephritis. Diagnosis is usually clinical (e.g., flank pain associated with nausea/vomiting, fever, pyuria, and bacteriuria), and no imaging technique is required. However, in the case of more complicated clinical pictures, ultrasound may be helpful [39]. Uncomplicated pyelonephritis can result in the kidney displaying a normal appearance. In the event of oedema or hemorrhage, areas that are hypoechoic or hyperechoic can be detected. The cortex and the medulla may lose their differentiation due to kidney enlargement [40]. Hypoechoic foci at the cortical–medullary junction can form in the kidney from time to time. These foci can develop as the infectious process progresses, indicating areas of bacterial nephritis. Large, well-defined hypoechoic masses that can sometimes be mistaken for cysts are the appearance of kidney abscesses [41].

Emphysematous pyelonephritis is a bacterial infection that is not common but could have an impact on diabetics. Using ultrasound, high-amplitude echoes can be observed in the renal parenchyma or sinus, and low-level echoes and reverberations can also be detected.

### 3.10. Masses and Cysts

**Figure 5:**
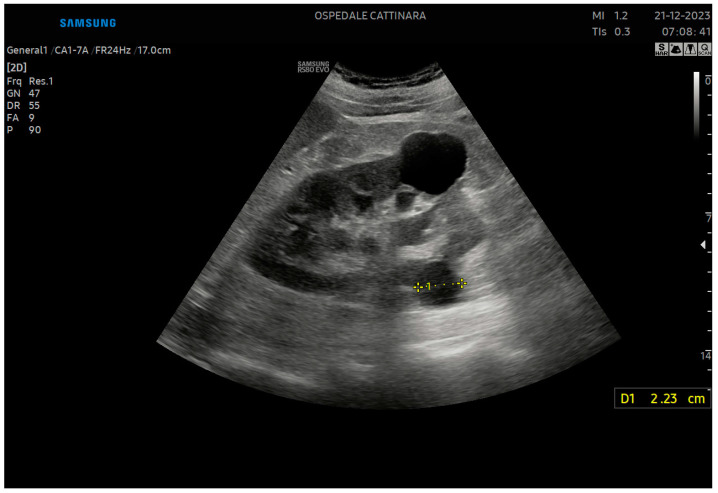
Two cysts of Bosniak 1 grade can be seen at the lower pole of the kidney. The Bosniak classification increases as the degree of sepimentation increases (complex cyst).

Although not related to acute medicine, the identification of masses and cysts necessitates initial characterization to select patients for in-depth diagnostic techniques. Cortical or parapelvic cysts are the most common types of renal cysts. Cortical cysts can either be intraparenchymal or exophytic and are typically found at the renal poles (Figure 5). They are anechogenic formations with regular margins, a thin wall, and acoustic reinforcement at the rear. Cysts can be unique or multiple, often bilateral, and of varying sizes. Until they reach remarkable dimensions and can compress contiguous structures, they are usually asymptomatic. Sometimes, they may have homogeneous echoes inside them (e.g., corpuscular content), single or multiple sepimentations, or parietal calcifications. The Bosniak classification distinguishes them into four grades based on the degree of complexity (e.g., sepimentation and solid content up to frank vegetations). The risk of malignant degeneration is progressively greater with increasing degrees.

The distinction between parapelvic cysts and hydronephrosis or lipomatosis of the renal sinus makes them important. The appearance is defined by a pattern of anechogenic formations that are arranged in rays near the cortical–medullary margin [42].

Ultrasound can be useful in confirming the diagnostic hypothesis of renal abscess, which is based on clinical symptoms like fever, flank pain, and leukocytosis. Clear contours, hypoechoic areas with extended anechogenic areas, and central colliquation are characteristic features of the renal abscess. In the color Doppler mode, there is no central vascular flow, but there is intense peripheral vascularization [42]. In the context of anaerobic bacterial infections, reverberant hyper-echogenic spots, which are referred to as gas bubbles, can be detected.

Malignant renal neoplasms account for 95% of renal neoplasms and originate from the parenchymal area. The most prevalent histotype is adenocarcinoma. Hematuria is often the initial clinical sign. Renal neoplasms appear as coarsely rounded masses of varied sizes that subvert the architecture of the parenchyma, extending from the cortical–medullary region into the calico-pelvic region. Carcinomas may appear as hypo-, iso-, or hyper-echogenic and exhibit intense vascularization in the color Doppler mode.

### 3.11. Acute Renal Venous Thrombosis

Patients may experience flank pain and tenderness, hypertension, and proteinuria as a result of acute renal venous thrombosis. Patients with kidney transplants have a high rate of venous thrombosis, but it should also be suspected in patients with nephrotic syndrome, malignancy, infections, and trauma. Ab-extrinsic compression (such as tumors, cysts, malformations, and aneurysms) is another reason to consider [43].

The endovascular thrombus can appear as a hyperechoic mass at times. A decrease in localized or generalized kidney echogenicity can be observed. The echo color Doppler scan results in the absence of parenchymal venous flow. A change in arterial flow can be observed, which could be caused by either the inversion of diastolic flow or an increased resistivity index above 0.75.

### 3.12. Renal Artery Stenosis

The presence of obstruction in either of the two renal arteries is typically accompanied by symptoms, such as a vascular murmur heard in the periumbilical area. Hypertension can appear in young individuals. In young individuals, renal artery stenosis may be linked to vasculitis or fibromuscular dysplasia or, more frequently (about 90%), to atherosclerosis. Early recognition of stenosis is crucial due to its progressive nature and potential for renal insufficiency. A high degree of diagnostic suspicion is necessary in cases where Sympathetic Crashing Acute Pulmonary Edema (SCAPE) is the initial manifestation [44]. In cases where a young adult has echocardiographic and electrocardiographic signs of arterial hypertension, it is recommended to have a bilateral renal artery study [45].

Occlusion of the renal artery or one of its main branches is evidenced by the absence of the main arterial color Doppler signal and/or a reduction in parenchymal arterial flows [46,47]. However, an echo color Doppler study may be of limited use in the case of obesity or overlying bowel gas, difficult/aberrant anatomy (e.g., horseshoe kidney, multiple renal arteries, and tortuous vessel), cardiac/aortic pulsation, and critically ill patients with difficulty following commands (e.g., extensive respiratory motion) [7].

### 3.13. Renal Resistivity Index

By using the color Doppler mode, the renal resistivity index (RRI), which is measured in the arcuate/interlobular arterioles, can be calculated as (peak systolic velocity−end diastolic velocity)/peak systolic velocity. The typical range is usually between 0.5 and 0.7.

**Figure 6:**
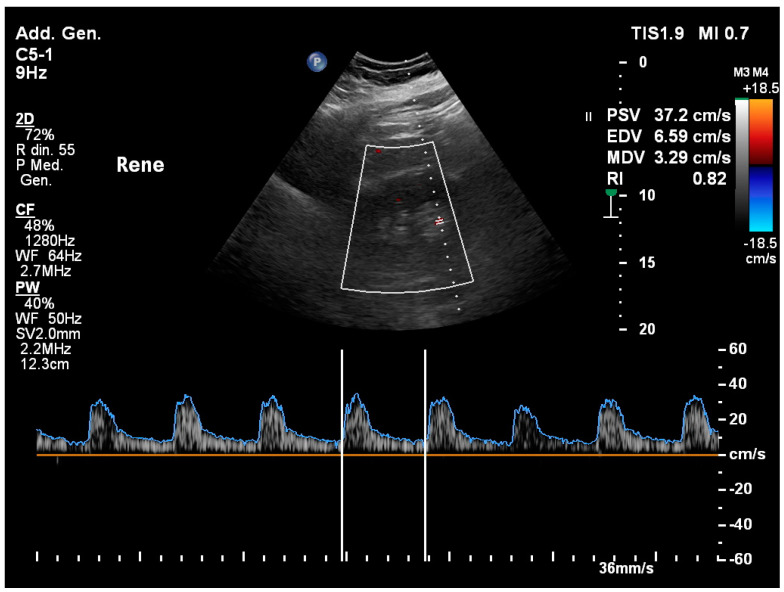
The renal resistance index, measured in the arcuate arteries (at the corticomedullary junction) or interlobar arteries (adjacent to medullary pyramids), allows for evaluation of the degree of resistance to intra-renal arterial flow.

The vascular and extravascular resistance of the renal parenchymal circle can be identified using this semi-quantitative parameter (Figure 6). The RRI is affected by vascular and parenchymal resistance downstream of the sphygmic wave sample in the afferent artery even when renal artery stenosis is not present. The RRI is affected by both parenchymal resistance and other intra-renal components, such as intra-renal compliance, which is determined by changes in venous and/or tubular-interstitial pressure. Other factors that contribute to extra-renal resistance (beyond renal artery stenosis) include changes in heart rate and systemic arterial compliance [48].

RRI can be used to evaluate kidney disease diagnosis and prognosis, including urinary obstruction, renal artery stenosis, diabetic nephropathy, and similar conditions. RRI has been shown to be an effective predictor of AKI reversibility in previous research. Meta-analyses found that a high RRI is associated with an increased risk of persistent AKI, with an AUC range between 0.83 and 0.92 compared to serum creatinine or oliguria [17,49]. However, caution should be taken in using RRI for this purpose, given the extreme heterogeneity of the population studied.

RRI has also been used as an indicator of the hemodynamic status of critically ill patients. It has been used as an early marker of shock [50], either to guide fluid management or to identify the optimal mean blood pressure during resuscitation [51]. However, even in these cases, several factors influencing the RRI prevent the guidelines from making recommendations.

### 3.14. Hemodinamyc Management Guided by Renal Ultrasound: The VExUS Protocol

**Figure 7:**
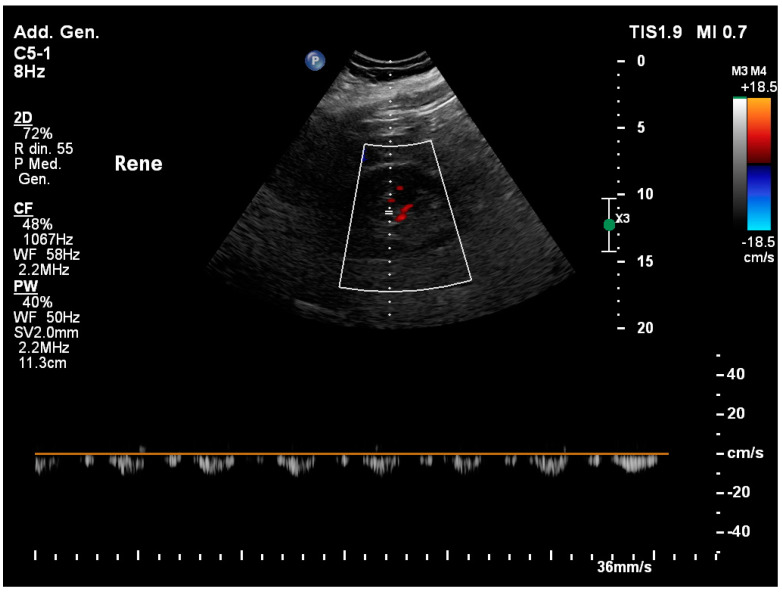
The diastolic phase is crucial in the ultrasound assessment of renal venous flow. When congestion occurs, the two components S and D turn discontinuous and pulsatile. At the most extreme levels of congestion, the two components are undistinguishable, like one single diastolic peak.

Decreased arterial perfusion and congestion of the renal vein affect the kidneys’ ability to compensate for the load of fluid. Renal congestion on the venous side is well documented to cause poor prognosis, but removing it early can improve kidney function and outcome. An overload or congestion condition can be determined using the color Doppler mode. To assess fluid status and make decisions about fluid management, ultrasounds can be performed on multiple organs, including the lungs, heart, inferior vena cava, internal jugular vein, and hepatic vein [52]. The color Doppler of intra-renal veins has been used to develop several indicators, including the venous impedance index, intra-renal venous flow, or renal venous stasis index. An increase in right atrial pressure can be caused by right heart failure or fluid overload. This increase in pressure affects the venous flow of the upstream organs (inferior vena cava, hepatic veins, portal veins, and renal veins). The hepatic vein usually displays a three-phase waveform because of the change in right atrial pressure (RAP) during the cardiac cycle. This waveform consists of the baseline, an A wave above the baseline (representing atrial systole), and two waveforms below the baseline (S and D) representing venous return during ventricular systole and diastole. In basal conditions, renal venous flow is a continuous flow. However, when congestion worsens, the flow becomes discontinuous (Figure 7). Waveform changes are divided into four flow patterns: continuous, discontinuous pulsatile, discontinuous biphasic (with venous peaks during systole and diastole), and discontinuous monophasic (with a venous peak during diastole) [52]. In addition to RAP, age, atherosclerosis, and intra-abdominal pressure are just a few of the factors that influence intracranial venous flow. It has recently been shown that monophasic discontinuous flow is related to AKI and poor prognosis.

A system that categorizes venous excess (VExUS) was established by Beaubien-Souligny et al. to assess venous congestion in cardiac surgery patients [53]. The VExUS classification system incorporates multiple IVC and Doppler flow patterns, including the hepatic vein, portal vein, and intra-renal vein. The severe VExUS grade is defined as dilated inferior vena cava (≥2 cm) and at least two severe abnormalities in the Doppler flow patterns (the presence of a reversed systolic phase in hepatic vein Doppler, pulsatility fraction >50% in portal vein Doppler, or a prevalent diastolic phase intra-renal venous Doppler).

### 3.15. Renal Trauma

Ultrasound is a foundational tool for detecting intraperitoneal fluid in hemodynamically unstable patients following trauma, with protocols like FAST and EFAST being widely implemented. While contrast-enhanced CT remains the gold standard for identifying lesions of the parenchyma, ultrasound is invaluable for ongoing monitoring, particularly for managing lesions conservatively without surgery. Furthermore, it can serve as a diagnostic method for assessing focal trauma in certain patient populations, such as children who are hemodynamically stable, demonstrating a sensitivity of 79% and specificity of 97% [54].

**Figure 8:**
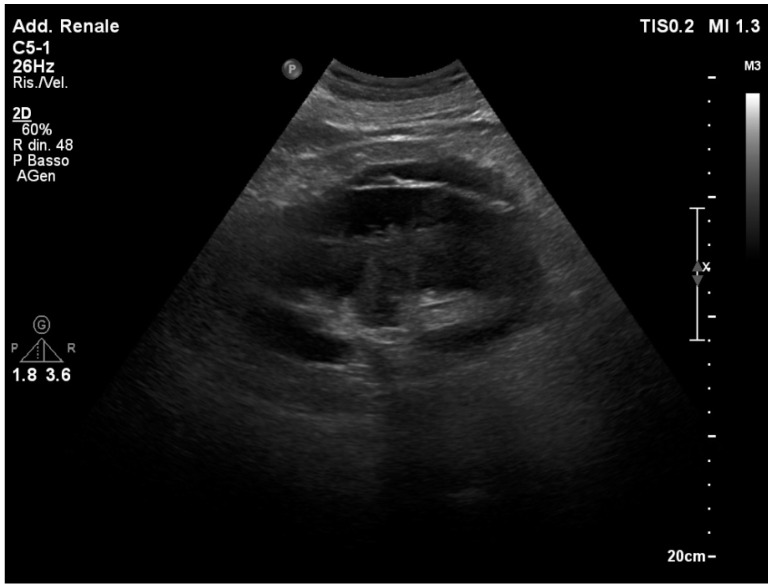
A hypoechoic area is visible in the presence of an extra-renal hematoma. The hematoma (hypoechoic area) has an intra-renal component that can be seen close to the cortical edge.

In cases involving the kidneys, ultrasound can reveal hypoechoic regions that correspond to localized parenchymal hemorrhage and oedema (Figure 8). It can also differentiate between a urinoma—which must be distinguished from a hematoma in situations where the urinary tract has ruptured—and fractures within the parenchyma. Additionally, color Doppler ultrasound is a useful adjunct for the detection of traumatic vascular injuries, which may include renal vein thrombosis.

### 3.16. Differential Diagnosis and Ruling out of Life-Threatening Diagnosis

Abdominal ultrasound serves as a crucial diagnostic tool in emergency medicine and critical care for detecting potentially fatal conditions. Even though certain illnesses may occur infrequently, they are nonetheless life-threatening. Clinicians employ bedside ultrasound in conjunction with traditional clinical signs to uncover serious pathologies like aortic dissection, bowel obstruction, abdominal aortic aneurysmal rupture risk, appendicitis, and other pertinent disorders [55]. In a significant multicenter study, Gibbons et al. evaluated the precision of integrated bedside ultrasound in diagnosing aortic dissection, demonstrating a 93% sensitivity and a 91% accuracy rate [56].

Demonstrating its clinical utility, abdominal ultrasound can guide the management of acute abdominal conditions, often expediting the decision for urgent surgical intervention. Remarkably, approximately 25% of patients requiring emergency or urgent surgery can receive a definitive diagnosis through bedside ultrasound alone, without the need for additional diagnostic procedures [57].

Ultrasound facilitates the etiological diagnosis of various pathologies and acute presentations. For instance, an ultrasound assessment of the kidneys during episodes of cardiac failure or hypertensive crises might unveil a pheochromocytoma as the underlying cause [58]. Such primary pheochromocytomas commonly originate in the adrenal glands (approximately 90%), though renal and vesical occurrences are also frequently observed [59].

### 3.17. Bladder Ultrasound in Emergency

Acute conditions like cystitis, urethritis, and prostatitis can lead to symptoms such as dysuria and bladder tenesmus. Oedema-induced diffuse or localized thickening of the bladder wall can be detected by ultrasound imaging. Chronic sub-vesical obstruction may result in morphological changes including the hypertrophy of the detrusor muscle, trabeculation of the bladder lining, and the formation of pseudodiverticula [60].

Conditions affecting the kidneys, such as lithiasis, can similarly impact the bladder. Stones may be identified in the bladder, urethra, and distal ureter, where they exhibit as hyperechoic formations casting a post-acoustic shadow with a twinkling artifact and mobility within the bladder upon patient repositioning [61].

**Figure 9:**
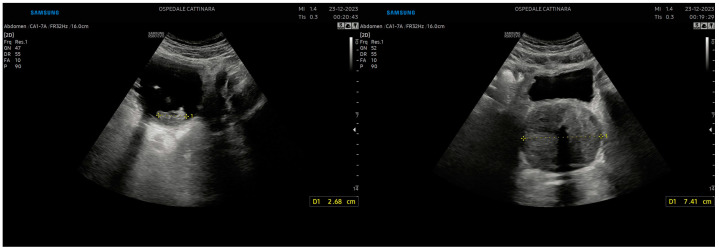
From left to right: Bladder tumors can take on a vegetative appearance, as in this case, or as a focal wall thickening. Benign prostatic hypertrophy, a condition that occurs after 40 years of age, can progress to degrees that can lead to the complete obstruction of urinary outflow in the urethra.

Neoplastic growths within the bladder can manifest as vegetative protrusions or as localized thickening of the bladder wall (Figure 9).

In men over the age of forty, benign prostatic hyperplasia is common and can lead to significant urinary obstruction in severe cases (Figure 9). In septic patients, acute prostatitis must be considered an infectious source, and ultrasound findings could include gland asymmetry, irregular borders, hypoechoic areas within the gland, and dilation of the peri-prostatic venous plexus [62].

### 3.18. Ultrasound-Guided Procedures for Genitourinary and Kidney System

Emergency and critical care healthcare professionals may perform genitourinary procedures where ultrasound guidance enhances procedural outcomes in both the ED and ICU. Ultrasound-guided suprapubic cystostomy boasts a high success rate while maintaining a low incidence of complications [63], proving especially effective in resource-constrained settings due to the portability and accessibility of ultrasound devices [64]. The importance of this is particularly evident in cases of acute urine retention and catheterization failure, or in patients with previous surgery or radiation at increased risk of bowel injury due to adhesions [63].

Percutaneous nephrostomy involves the catheterization of the renal pelvis and is indicated for cases of acute urinary obstruction with significant hydronephrosis. This approach is particularly pertinent if the patient exhibits symptoms or shows impending signs of renal failure. It is also indicated for urinary tract infections such as pyelonephritis with accompanying pyonephrosis. While nephrostomy tubes can be positioned under CT scan, fluoroscopic, or ultrasound guidance, the success rates for fluoroscopic and ultrasound guidance are comparable. However, the ultrasound-guided approach tends to result in fewer complications [65]. Given its advantage of being ionizing radiation-free, sonography is the preferred method for certain populations, including pediatric and pregnant patients [66].

Procedural ultrasound is also capable of aiding urinary catheterization. It allows for the inspection of the ultrasound anatomy of the bladder before the procedure, provides an estimation of its residual urine volume, and can detect the progression of the urinary catheter into the urethra [67]. By contrast, blind catheterization is associated with a higher risk of (a) inappropriate catheterization, (b) unsuccessful catheterization, (c) traumatic injuries, and (d) risk of infection [68]. A pre–post study performed in 2023 [69] found that ultrasound-guided catheterization significantly reduced the rate of inappropriate catheterization (from 22.6% in the pre-group, to no inappropriate catheterization in the post-group), causing a 2.2% absolute reduction in the rate of urinary tract infections (from 8.5% to 6.3%) and costs associated with the use of urinary catheters (by 74.2%). The study concluded that bladder ultrasound in clinical practice is feasible and reduces the inappropriate use of bladder catheters, being capable of reducing patient risks and inappropriate healthcare costs [69]. Echo-guided urethral catheterization is particularly indicated in patients with known or suspected urethral restrictions or in cases of difficult catheterization [70].

## 4. Discussion

This overview summarized the ultrasound evaluation of the renal and genitourinary systems, providing a summary that can be utilized as a guide by emergency and critical care healthcare providers for both diagnostic and procedural scopes. We described the role of ultrasound in investigating several conditions such as AKI, renal colic, ureteral stones, the ureteral jet, ureteropelvic junction obstructions, acute renal infections, the presence of masses and cysts, acute renal thrombosis, renal artery stenosis, the renal resistivity index, renal trauma, as well as the differential diagnosis of life-threatening conditions and the ultrasound-guided hemodynamic management and procedures.

Ultrasound is rising and becoming acknowledged as a first-line imaging resource [71], thus its role in managing acute nephropathy and genitourinary issues is increasingly crucial given its immediate bedside availability, which is particularly beneficial in settings with limited resources [72].

This imaging modality not only facilitates the initiation of therapeutic interventions but also provides swift prognostic insights that are vital to tailoring patient care. In AKI cases, ultrasound is instrumental for determining the cause of renal failure [73]. Moreover, it provides rapid assessment of the patient’s hemodynamic status, informing and guiding therapeutic decisions [74].

A wider use of ultrasound is advised [75] and it is recommended that medical and healthcare professions define a set of minimum requirements for the equipment and education [76]. The increase in usage of POC ultrasound techniques will be also aided by the affordability, availability, portability, and durability of ultrasound machines [77]. In parallel with the introduction of novel hardware and equipment technologies, the diffusing implementation of Artificial Intelligence (AI) in ultrasound [71] may have the potential to provide several advantages: from fostering healthcare professionals’ confidence in diagnosis [71] to automatic measurements and automatic visual pattern recognition.

## 5. Conclusions

Renal and genitourinary ultrasound has been proven to be effective in several emergency and critical care scopes, assisting healthcare professionals at the bedside in diagnosis and procedures. As further advances in ultrasound will arise, it is important for healthcare providers to foster the use of these technologies capable of improving patient outcomes [78].

## Data Availability

Not applicable.
